# Prolonged Depression-Like Behavior Caused by Immune Challenge: Influence of Mouse Strain and Social Environment

**DOI:** 10.1371/journal.pone.0020719

**Published:** 2011-06-06

**Authors:** Evelin Painsipp, Martin J. Köfer, Frank Sinner, Peter Holzer

**Affiliations:** 1 Research Unit of Translational Neurogastroenterology, Institute of Experimental and Clinical Pharmacology, Medical University of Graz, Graz, Austria; 2 Health-Institute for Biomedicine and Health Sciences, Joanneum Research, Graz, Austria; University of Wuerzburg, Germany

## Abstract

Immune challenge by bacterial lipopolysaccharide (LPS) causes short-term behavioral changes indicative of depression. The present study sought to explore whether LPS is able to induce long-term changes in depression-related behavior and whether such an effect depends on mouse strain and social context. LPS (0.83 mg/kg) or vehicle was administered intraperitoneally to female CD1 and C57BL/6 mice that were housed singly or in groups of 4. Depression-like behavior was assessed with the forced swim test (FST) 1 and 28 days post-treatment. Group-housed CD1 mice exhibited depression-like behavior 1 day post-LPS, an effect that leveled off during the subsequent 28 days, while the behavior of singly housed CD1 mice was little affected. In contrast, singly housed C57BL/6 mice responded to LPS with an increase in depression-like behavior that was maintained for 4 weeks post-treatment and confirmed by the sucrose preference test. Group-housed C57BL/6 mice likewise displayed an increased depression-like behavior 4 weeks post-treatment. The behavioral changes induced by LPS in C57BL/6 mice were associated with a particularly pronounced rise of interleukin-6 in blood plasma within 1 day post-treatment and with changes in the dynamics of the corticosterone response to the FST. The current data demonstrate that immune challenge with LPS is able to induce prolonged depression-like behavior, an effect that depends on genetic background (strain). The discovery of an experimental model of long-term depression-like behavior after acute immune challenge is of relevance to the analysis of the epigenetic and pathophysiologic mechanisms of immune system-related affective disorders.

## Introduction

Experimental and clinical evidence indicates that activation of the immune system contributes to the pathogenesis of mood disorders [1]–[6]. Patients with major depression have frequently been observed to present with elevated levels of proinflammatory cytokines in blood plasma and cerebrospinal fluid [4]. There is extensive comorbidity of major depression with medical conditions involving inflammation and an increased expression of cytokines, and the therapeutic use of cytokines such as interferons is known to induce a depression-like syndrome in a sizeable proportion of patients [5]. These lines of clinical evidence are complemented by a plethora of animal studies. Both peripheral induction of cytokines by infection [6]–[9] or cancer [10] and intracerebral administration of cytokines to rodents evoke depression-like symptoms which are abrogated by cytokine antagonists or cytokine synthesis blockers.

Peripheral induction of cytokines by systemic administration of bacterial lipopolysaccharide (LPS) at doses that are too low to evoke a shock-like condition is known to induce a behavioral syndrome that includes traits of depression and follows a distinct time course [3], [6], [11]. Initially, a response termed “sickness behavior” is prevailing, which includes fever, anorexia, reduction of locomotion and a decrease in social interaction. Once the sickness behavior in terms of anorexia and sedation is over, behavioral symptoms indicative of depression such as anhedonia and passive stress coping are observed 24–48 h post-treatment [12], [13]. When neuropeptide Y receptors of subtype Y2 or Y4 have been knocked out, depression-like behavior is seen even 4 weeks after a single injection of low-dose LPS [14]. This observation led us to hypothesize that, depending on genetic background (mouse strain) and social context (single versus group housing), intraperitoneal (IP) injection of LPS is able to induce long-term depression-like behavior. We addressed this hypothesis by studying the behavioral effect of LPS with the forced swim test (FST), a measure of behavioral despair [15], and the sucrose preference test, a measure of depression-related anhedonia [12]. These tests were carried out 1 day and 4 weeks post-LPS treatment.

Two strains of mice, outbred CD1 mice and inbred C57BL/6 mice, were compared with each other. CD1 mice were selected because short-term behavioral changes indicative of a state of depression following LPS treatment have extensively been studied in this mouse strain [12]. C57BL/6 mice were chosen because the Y2 and Y4 receptor knockout mice that exhibit long-term depression-like behavior following LPS treatment have a 50% C57BL/6 background [14]. In addition, C57BL/6 mice harbor a serotonin transporter haplotype defined by two non-synonymous coding variants, which have implications on serotonin transporter function [16]. The experiments were carried out with female mice, given that affective disorders are more prevalent in women than in men [17] and there is a need to overcome the sex bias that is present in the neurosciences [18].

Apart from genetic background, psychosocial context may be another factor relevant to the manifestation of mood disturbances due to immune challenge [19]–[22]. Psychosocial stress is able to evoke cerebral expression of cytokines [5], [23], [24], and prolonged separation of mice enhances the LPS-evoked sickness behavior [22]. Since social isolation of mice is also able to enhance depression-like behavior [25], we used this experimental paradigm to address the question whether social context modifies the effect of LPS to induce long-term changes in affective behavior. Thus, female CD1 and C57BL/6 mice were either kept in groups of 4 or housed singly throughout the course of the experiments.

LPS-evoked peripheral immune challenge alters brain functions by a neural and an endocrine route [6], [11]. In addition, LPS and proinflammatory cytokines are able to stimulate the hypothalamic-pituitary-adrenal (HPA) axis as revealed by a rise of circulating glucocorticoid levels [4]–[6], [22]. For this reason, plasma levels of interleukin-6 and corticosterone were monitored to examine whether any behavioral changes are correlated with these factors.

## Materials and Methods

### Experimental animals

The study was carried out with age-matched, adult, 4–6 month old, female mice of the outbred strain CD1 and the inbred strain C57BL/6, which were obtained from Charles River Laboratories (Sulzfeld, Germany). The animals were kept in groups of 4 in cages of size IIL (length×width×height = 26 cm×20.5 cm×14 cm) in the institutional animal house in which the temperature (set point 22°C), relative air humidity (set point 50%) and light conditions (lights on at 6:00 h, lights off at 18:00 h, maximal intensity 150 lux) were tightly controlled. Tap water and standard laboratory chow were provided ad libitum throughout the study.

### Ethics statement

The experimental procedures and number of animals used in this study were approved by an ethical committee at the Federal Ministry of Science and Research of the Republic of Austria and conducted according to the Directive of the European Communities Council of 24 November 1986 (86/609/EEC). The experiments were designed in such a way that the number of animals used and their suffering was minimized.

### Administration of lipopolysaccharide (LPS)

LPS extracted from *E. coli* 0127:B8 (purified by gel-filtration chromatography, catalogue number L3137, Sigma-Aldrich, Vienna, Austria) was dissolved in pyrogen-free sterile saline (0.9% NaCl) at a concentration of 1 mg/ml. This stock solution was diluted with pyrogen-free sterile saline to yield an injection solution of 0.083 mg/ml LPS, which was injected IP at a volume of 0.01 ml/g, equivalent to a dose of 0.83 mg/kg LPS [12], [14]. Pyrogen-free sterile saline injected at the same volume was used as vehicle control.

### Experimental groups and time lines

The experiments were started after the animals had become familiar with the institutional animal house over the course of at least 3 weeks. Prior to the behavioral tests, the mice were allowed to adapt to the test room (set points 22°C and 50% relative air humidity, lights on at 6:00 h, lights off at 18:00 h, maximal light intensity 100 lux) for at least two days.

**Figure 1:**
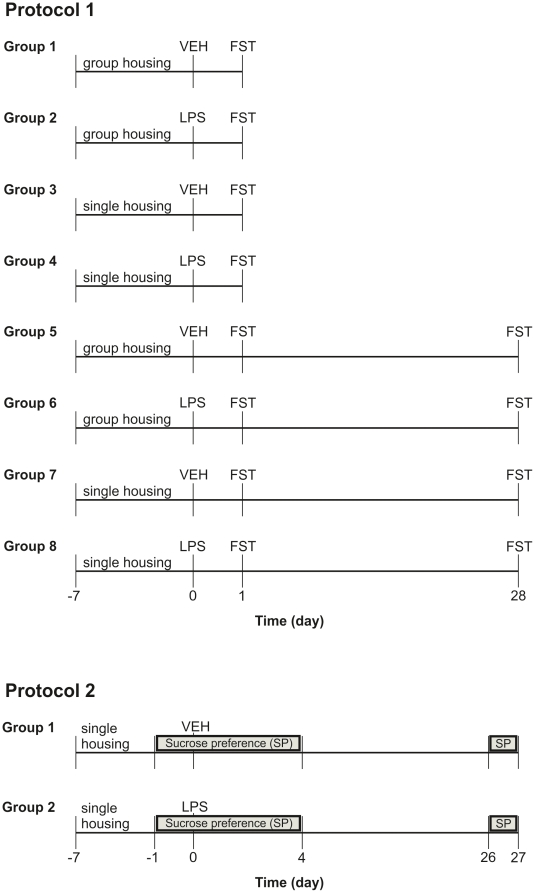
Experimental groups and time lines. In protocol 1, the effect of IP injected vehicle (VEH) and LPS on depression-like behavior in the FST was assessed 1 and 28 days post-injection. In protocol 2, the depression-related effect of vehicle and LPS was assessed with the sucrose preference (SP) test during days −1–4 and days 26–27 post-injection.

Two different protocols were used (Figure 1). In protocol 1, the effect of IP injected vehicle and LPS on depression-like behavior in the FST was assessed 1 and 28 days post-injection. For this purpose a total of 64 CD1 and 64 C57BL/6 mice was employed. The animals of each strain were allocated to 8 experimental groups (Figure 1), each group comprising 8 mice. The animals were either kept in sets of 4 animals per cage (group housing) or housed individually (single housing). The single housing protocol was started 7 days before IP injection of vehicle or LPS (Figure 1). After the FST, the mice were instantly returned to their home cage, and care was taken not to change cage mates during the course of the experiment. Thirty minutes after the start of the FST, trunk blood was collected for determination of the circulating levels of corticosterone and interleukin-6.

In protocol 2, the effect of vehicle and LPS on the sucrose preference of 14 singly housed C57BL/6 mice was assessed during days −1–4 and days 26–27 post-treatment (Figure 1). The single housing protocol was started 7 days before IP injection of vehicle or LPS. The animals were offered the choice to drink from two bottles, one filled with tap water and the other filled with 1% sucrose in tap water. The daily intake of tap water and sucrose solution was estimated by weighing the bottles every day.

### Forced swim test (FST)

Each mouse was placed individually in a glass cylinder (diameter: 16 cm, height: 23 cm) containing tap water at 25°C. The water was 16 cm deep, which prevented the mice from touching the bottom of the beaker with their paws or the tail. Three categories of behavioral activity (climbing, swimming, and immobility) during the 6 min test session [14], [26] were scored by a trained observer. The time each mouse spent climbing, swimming and floating (immobile) during the FST was recorded and expressed as a percentage of the test duration. Mice were considered immobile when they floated passively in the water, performing only movements that enabled them to keep their heads above the water level [15]. After the FST, the mice were placed for 6 min under a warming lamp and then returned to their home cage.

### Sucrose preference

The animals were offered the choice to drink from two bottles, one filled with tap water and the other filled with 1% sucrose in tap water. The daily intake of tap water and sucrose solution was estimated by weighing the bottles every day between 8:30 h and 9:00 h. After they had been weighed, the bottles were cleaned and refilled every day. In addition, the relative position of the two bottles in the cage lid was changed every day.

### Circulating corticosterone

Thirty minutes after the FST, between 11:00 h and 13:00 h, mice were deeply anesthetized with pentobarbital (150 mg/kg IP) before they were decapitated. Within 2 min after the injection of anesthetic, trunk blood was collected into vials coated with ethylenediamine tetraacetate (Greiner, Kremsmünster, Austria) kept on ice. Following centrifugation for 10 min at 4°C and 1200×g, blood plasma was collected and stored at −70°C until assay. The plasma levels of corticosterone were determined with an enzyme immunoassay kit (Assay Designs, Ann Arbor, Michigan, USA). According to the manufacturer's specifications, the sensitivity of the assay is 27 pg/ml, and the intra- and inter-assay coefficient of variation amounts to 7.7 and 9.7%, respectively.

### Circulating interleukin-6

A part of the blood plasma collected for determination of corticosterone was used for the assay of circulating interleukin-6. The plasma levels of interleukin-6 were determined with an enzyme immunoassay kit (Fluorokine MAP Mouse IL-6 Kit, R&D Systems, Minneapolis, Minnesota, USA). According to the manufacturer's specifications, the sensitivity of the assay is 1.1 pg/ml, and the intra- and inter-assay coefficient of variation amounts to 4.0 and 7.4%, respectively.

### Statistics

Statistical evaluation of the results was made with SPSS 16.0 (SPSS Inc., Chicago, Illinois, USA). In general, the data were analyzed by two-way analysis of variance (ANOVA), in some cases for repeated measurements. The homogeneity of variances was assessed with the Levene test. In case of sphericity violations the Greenhouse-Geisser correction was applied. Post-ANOVA analysis of group differences was performed with the Tukey HSD (honestly significant difference) test, when the variances were homogeneous, and with the Games-Howell test, when the variances were unequal. Student's *t* test was used when only two data groups were compared with each other. Some experiments were analyzed by *planned comparisons* with the Mann-Whitney test [27]. In view of the exploratory nature of the study, probability values *P*≤0.1 [27]–[29] were regarded as statistically significant. All data are presented as means±SEM, n referring to the number of mice in each group.

## Results

### LPS reduced body weight 1 day post-treatment

**Figure 2:**
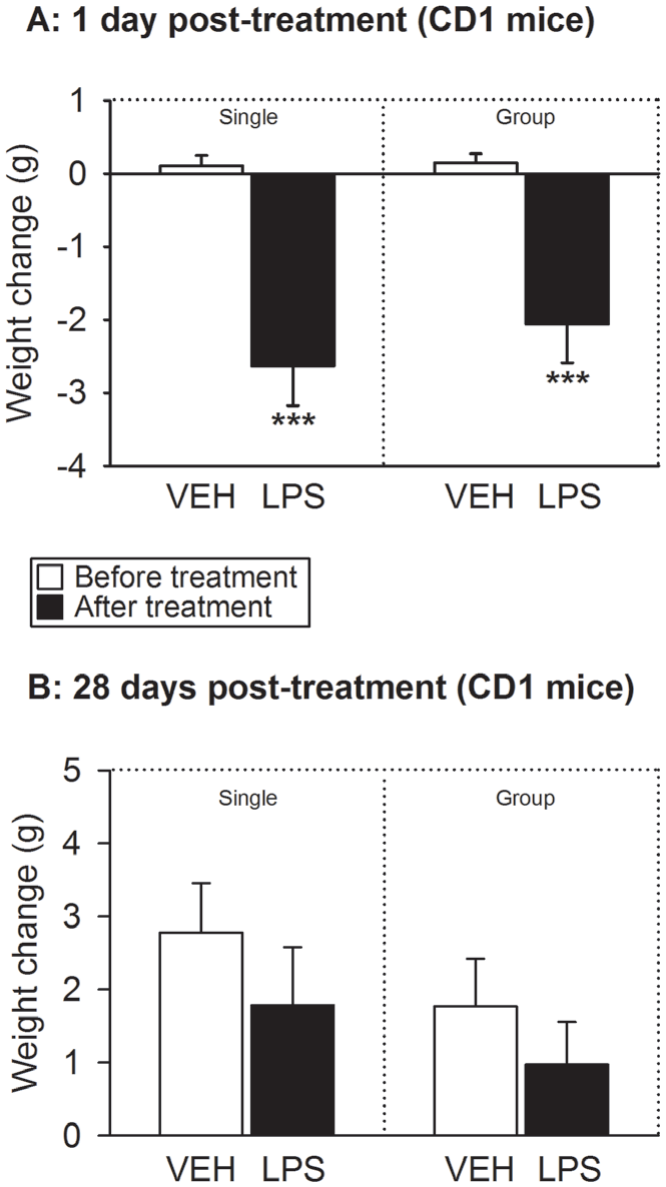
Effect of LPS (0.83 mg/kg injected IP), relative to vehicle (VEH), on the body weight of CD1 mice as measured immediately before and 1 day (A) as well as 28 days (B) after treatment under single and group housing conditions. The graphs show the change in weight (weight after treatment minus weight before treatment). The values are means+SEM, n = 8. *** *P*<0.01 versus change in weight following treatment with vehicle.

**Figure 3:**
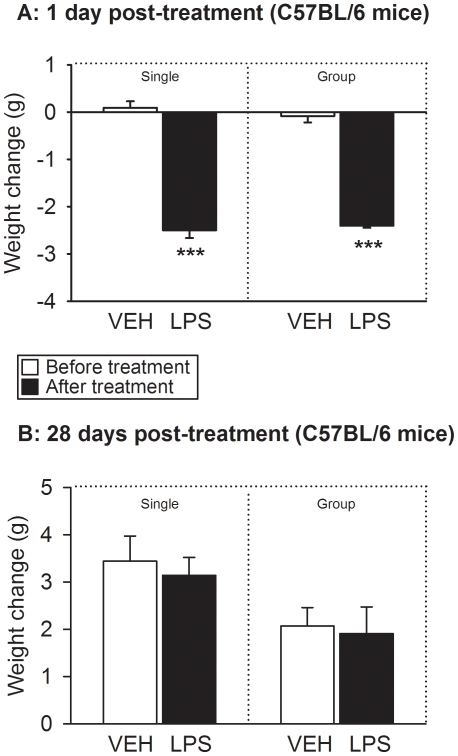
Effect of LPS (0.83 mg/kg injected IP), relative to vehicle (VEH), on the body weight of C57BL/6 mice as measured immediately before and 1 day (A) as well as 28 days (B) after treatment under single and group housing conditions. The graphs show the change in weight (weight after treatment minus weight before treatment). The values are means+SEM, n = 7–8. *** *P*<0.01 versus change in weight following treatment with vehicle.

The body weight of the animals was recorded immediately before (day 0) and 1 day after IP injection of vehicle or LPS. The body weight before treatment did not differ between the experimental groups of CD1 and C57BL/6 mice under study. Specifically, the baseline weight of the CD1 mice was 31.6±0.34 g (n = 64) and that of C57BL/6 mice 19.1±0.10 g (n = 64), which is typical of these strains. Treatment with LPS reduced the body weight of CD1 (Figure 2A) and C57BL/6 (Figure 3A) mice to a significant extent, while vehicle treatment had no effect. The LPS-induced decrease in body weight was on average 2.1–2.6 g in CD1 mice and 2.4–2.5 g in C57BL/6 mice (Figures 2A and 3A). In both strains of mice this weight loss did not significantly differ between singly housed and group-housed animals (Figures 2A and 3A). The detailed results of the statistical analysis of the data are reported in Text S1.

### LPS had no effect on body weight 28 days post-treatment

When CD1 and C57BL/6 mice were weighed 28 days post-treatment, their body weight was higher than immediately before IP injection of LPS or its vehicle (Figures 2B and 3B). The increase in body weight amounted on average to 1.0–2.8 g in CD1 mice and to 1.9–3.4 g in C57BL/6 mice (Figures 2B and 3B) and was statistically significant in either strain of mice (see Text S1). However, the weight gain did not significantly differ between singly housed and group-housed animals and between vehicle- and LPS-treated mice of either strain (Figures 2B and 3B).

### LPS induced short-term depression-like behavior in group-housed CD1 mice

**Figure 4:**
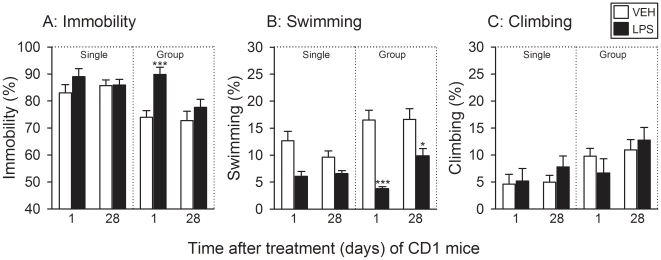
Effect of LPS (0.83 mg/kg injected IP), relative to vehicle (VEH), on the behavior of CD1 mice in the FST as recorded 1 and 28 days after treatment under single and group housing conditions. The graphs show (A) the duration of immobility, (B) the duration of swimming, and (C) the duration of climbing, these parameters being expressed as a percentage of the total test duration. The values are means+SEM, n = 8. * *P*≤0.1, *** *P*<0.01 versus vehicle-treated mice at the same time point post-treatment. In panel B (Single) it was not possible to apply a post-hoc test because two-way ANOVA failed to disclose any interaction between the factors time and treatment.

The behavior in the FST was assessed 1 and 28 days after IP injection of LPS or its vehicle and analyzed with respect to the factors time (1 day versus 28 days post-treatment) and treatment (LPS versus vehicle). Relative to vehicle, LPS failed to alter the duration of immobility and climbing in singly housed CD1 mice both 1 day and 4 weeks after treatment (Figure 4A,C). In contrast, the duration of time spent swimming by singly housed CD1 mice was significantly reduced by LPS (see Text S1), but since there was no interaction between the factors time and treatment, this effect of LPS could not be further analyzed (Figure 4B).

The effect of LPS on the FST behavior in group-housed CD1 mice was much more pronounced than in singly housed CD1 mice (Figure 4). Relative to vehicle, LPS significantly prolonged the duration of immobility in group-housed CD1 mice 1 day, but not 28 days, post-treatment (Figure 4A). This LPS-induced extension of immobility was complemented by a highly significant shortening of the duration of swimming 1 day post-treatment, whereas 28 days post-treatment the effect of LPS to reduce the duration of swimming in group-housed CD1 mice was clearly less pronounced but still statistically significant (Figure 4B). In contrast, the duration of climbing spent by group-housed CD1 mice was not significantly altered by LPS 1 and 28 days post-treatment (Figure 4C).

### LPS induced short- and long-term depression-like behavior in C57BL/6 mice

**Figure 5:**
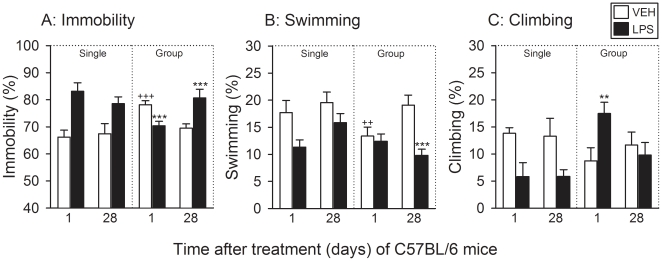
Effect of LPS (0.83 mg/kg injected IP), relative to vehicle (VEH), on the behavior of C57BL/6 mice in the FST as recorded 1 and 28 days after treatment under single and group housing conditions. The graphs show (A) the duration of immobility, (B) the duration of swimming, and (C) the duration of climbing, these parameters being expressed as a percentage of the total test duration. The values are means+SEM, n = 8. ** *P*<0.05, *** *P*<0.01 versus vehicle-treated mice at the same time point post-treatment, ++ *P*<0.05, +++ *P*<0.01 versus vehicle-treated mice tested 28 days post-treatment. In panels A (Single), B (Single) and C (Single) it was not possible to apply a post-hoc test because two-way ANOVA failed to disclose any interaction between the factors time and treatment.

The experiments conducted with C57BL/6 mice were analogous to those carried out with CD1 mice, yet the outcome of the FST differed substantially between CD1 mice (Figure 4) and C57BL/6 mice (Figure 5). Two-way ANOVA disclosed an effect of treatment (LPS versus vehicle) on the duration of immobility, swimming and climbing (see Text S1). However, since there was no significant interaction between the factors treatment and time, the effect of LPS could not be subjected to post-hoc analysis of group differences. Notwithstanding this fact, LPS prolonged the duration of immobility in singly housed C57BL/6 mice (Figure 5A), while at the same time the duration of climbing was shortened (Figure 5C). In addition, the duration of swimming was reduced by LPS in a time-dependent manner (Figure 5B). A synopsis of the data obtained in singly housed C57BL/6 mice indicates that LPS prolonged the duration of immobility along with a shortening of the duration of both swimming and climbing, these changes being numerically more pronounced 1 day than 28 days post-treatment (Figure 5A,B,C).

The effect of LPS on the behavior of group-housed C57BL/6 mice in the FST showed a distinct time course over the 28-day period post-treatment. Thus, the duration of immobility was significantly shortened by LPS 1 day, but significantly prolonged 28 days post-treatment (Figure 5A). In addition, the duration of immobility recorded in vehicle-treated animals 1 day post-treatment was significantly longer than that recorded 28 days post-treatment (Figure 5A). The changes in immobility duration which LPS evoked in group-housed C57BL/6 mice (Figure 5A) were complemented by alterations in the duration of swimming (Figure 5B) and climbing (Figure 5C). Specifically, LPS shortened the duration of swimming in group-housed C57BL/6 mice 28 days post-treatment, but not 1 day post-treatment (Figure 5B). In addition, the duration of swimming recorded in vehicle-treated animals 1 day post-treatment was shorter than that recorded 28 days post-treatment (Figure 5B). The duration of climbing observed in group-housed C57BL/6 mice was significantly prolonged by LPS 1 day, but not 28 days, after treatment (Figure 5C). Taken all aspects together, it is evident that in group-housed C57BL/6 mice LPS shortened immobility 1 day post-treatment due to an increase in climbing, but prolonged immobility 28 days post-treatment at the expense of swimming (Figure 5A,B,C).

### LPS caused short-term, but not long-term, increases in circulating corticosterone levels

**Figure 6:**
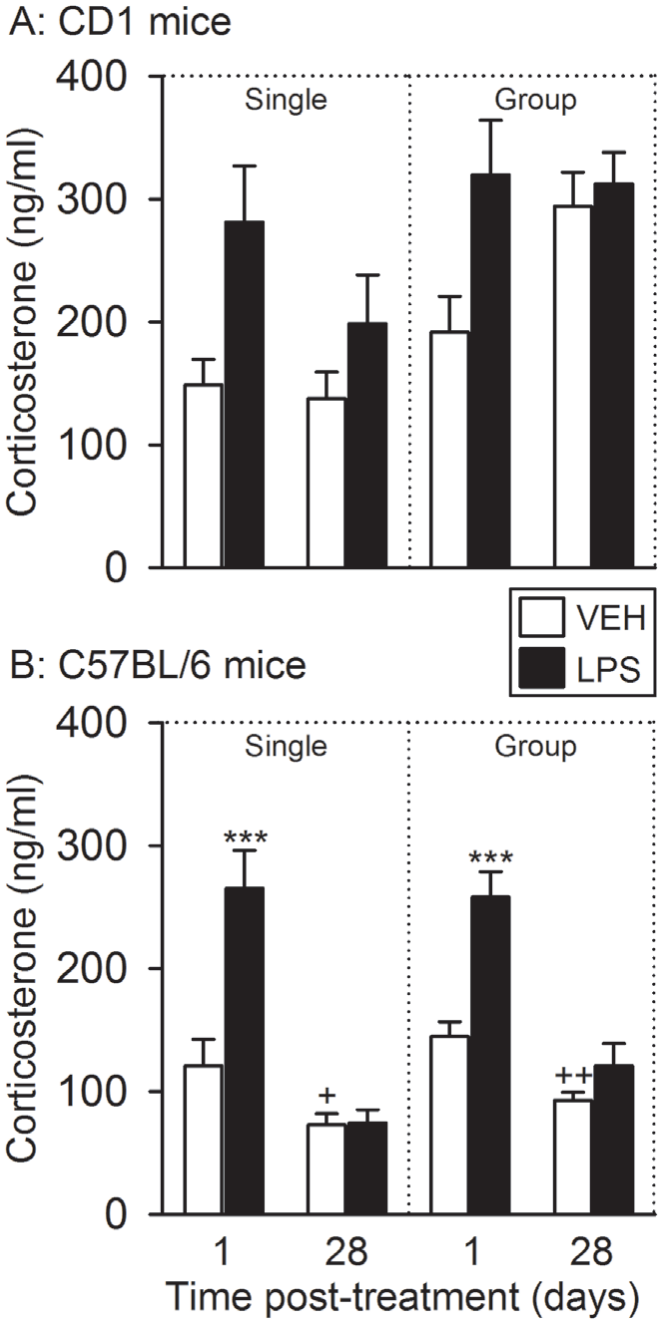
Effect of LPS (0.83 mg/kg injected IP), relative to vehicle (VEH), on plasma concentrations of corticosterone as recorded 1 and 28 days after treatment under single and group housing conditions. Trunk blood of CD1 (A) and C57BL/6 (B) mice for the corticosterone assay was taken 30 min after the FST had been started. The values are means+SEM, n = 7–8. *** *P*<0.01 versus vehicle-treated mice at the same time point post-treatment, + *P*≤0.1, +++ *P*<0.01 versus vehicle-treated mice tested 1 day post-treatment. In panel A it was not possible to apply a post-hoc test because two-way ANOVA failed to disclose any interaction between the factors time and treatment.

The plasma concentrations of corticosterone were measured 30 min after the FST had begun. Two-way ANOVA of the corticosterone levels measured in singly housed CD 1 mice (Figure 6A) revealed an effect of treatment (LPS versus vehicle) but, since there was no significant interaction between the factors treatment and time (see Text S1), the effect of LPS could not be subjected to post-hoc analysis of group differences. Despite this fact it is evident from Figure 6A that LPS enhanced the post-FST levels of corticosterone in singly housed CD 1 mice and that this LPS-induced increase in circulating corticosterone was nominally more pronounced 1 day post-treatment than 28 days post-treatment (Figure 6A). A similar result was obtained in group-housed CD1 mice (see Text S1) in which the effect of LPS to enhance the post-FST plasma corticosterone levels was seen 1 day, but not 28 days, post-treatment (Figure 6A).

The effect of LPS on the post-FST plasma concentrations of corticosterone in C57BL/6 mice was analogous to that seen in CD1 mice. One day post-treatment, LPS evoked a comparable rise of circulating corticosterone in both singly housed and group-housed C57BL/6 mice, an effect that was no longer observed 28 days post-treatment (Figure 6B). In addition, the post-FST plasma levels of corticosterone measured 28 days after vehicle treatment of singly housed and group-housed C57BL/6 mice were significantly lower than 1 day after vehicle treatment (Figure 6B).

Additional analysis revealed that the post-FST plasma concentrations of corticosterone depended on the housing conditions. Thus, the circulating levels of the glucocorticoid measured 28 days after vehicle/LPS treatment of singly housed CD1 and C67BL/6 mice were lower than those in the respective group-housed animals (Figure 6A,B).

### The effect of LPS to transiently increase circulating interleukin-6 levels was more pronounced in C57BL/6 mice than in CD1 mice

**Figure 7:**
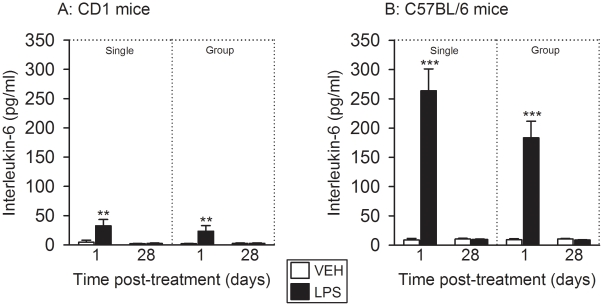
Effect of LPS (0.83 mg/kg injected IP), relative to vehicle (VEH), on plasma concentrations of interleukin-6 as recorded 1 and 28 days after treatment under single and group housing conditions. Trunk blood of CD1 (A) and C57BL/6 (B) mice for the interleukin-6 assay was taken 30 min after the FST had been started. The values are means+SEM, n = 7–8. ** *P*<0.05, *** *P*<0.01 versus vehicle-treated mice at the same time point post-treatment.

The plasma levels of interleukin-6 were measured 30 min after the FST had begun. Relative to vehicle, LPS increased the circulating concentrations of interleukin-6 1 day, but not 28 days, after treatment of singly housed and group-housed CD1 mice (Figure 7A). C57BL/6 mice turned out to be particularly sensitive to the acute effect of LPS inasmuch as the plasma concentrations of interleukin-6 measured 1 day after LPS treatment were much higher than in CD1 mice (Figure 7A,B). While, in CD1 mice, LPS enhanced the plasma concentrations of interleukin-6 on average by factors of 7.2–12.1, it increased circulating interleukin-6 in C57BL/6 mice on average by factors of 20.2–30.5 (Figure 7A,B). Much as in CD1 mice, however, the effect of LPS had waned 28 days after treatment of singly housed and group-housed C57BL/6 mice (Figure 7B). In neither strain of mice did the interleukin-6 concentrations significantly differ between singly housed and group-housed animals.

### LPS caused a long-term inhibition of sucrose preference in C57BL/6 mice

**Figure 8:**
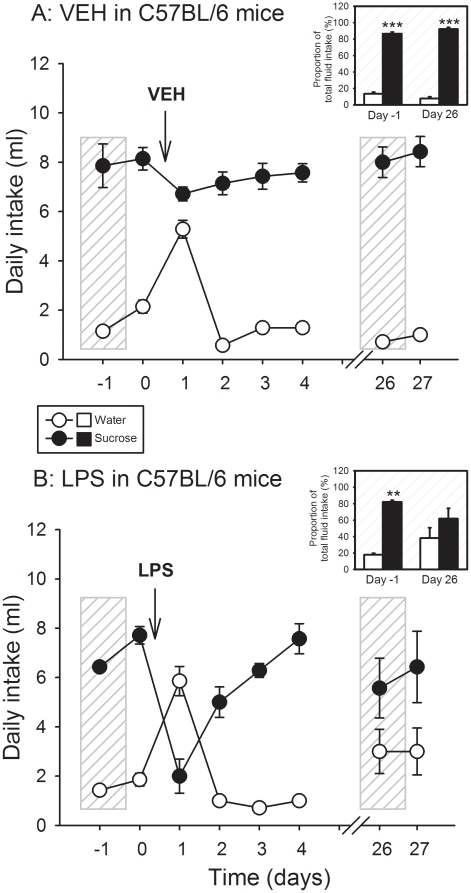
Effect of (A) vehicle (VEH) and (B) LPS (0.83 mg/kg injected IP) on the daily consumption of sucrose (1%) solution and normal tap water in singly housed C57BL/6 mice. The main graphs show the absolute daily intake of sucrose solution and water (ml). In the inserts, the daily intake of sucrose solution and water on the day before treatment (day -1) and on day 26 post-treatment is expressed as a percentage of the total daily fluid intake. The values are means±SEM, n = 7. *** *P*<0.01 versus water intake.

The effect of LPS on sucrose preference and total water intake was investigated in singly housed C57BL/6 mice for a period of 27 days. As expected, the animals strongly preferred sucrose (1%) solution over normal tap water (Figure 8A,B). Following injection of vehicle or LPS, there was a change in their drinking behavior. Vehicle treatment transiently increased the consumption of water and slightly decreased the intake of sucrose solution during the 24-h period post-treatment (Figure 8A). Planned comparison of the relative contribution of sucrose solution and water intake to the total consumption of fluid revealed that on day 26 after vehicle treatment the relative intake of sucrose solution and water was indistinguishable from that measured during the day before vehicle treatment (Figure 8A, insert). Both on day 1 before (day -1) and on day 26 after vehicle treatment, the intake of sucrose solution covered more than 80% of the total consumption of fluid (Figure 8A, insert).

In contrast to vehicle, LPS led to a short-term reversal of sucrose preference to water preference. As is shown in Figure 8B, the intake of sucrose solution was reduced by more than 70% during the 24-h period after LPS treatment, while the consumption of water was enhanced. The relative consumption of sucrose solution versus water normalized over the following days, but on days 26 and 27 post-treatment the relative intake of sucrose solution was nominally lower, and that of water nominally higher, than before LPS treatment (Figure 8B). This instance was also revealed by planned comparison of the relative contribution of sucrose solution and water intake to the total consumption of fluid (Figure 8B, insert). While on day 1 before LPS treatment (day -1) the intake of sucrose solution covered some 80% of the total consumption of fluid, the relative intake of sucrose solution and water was no longer significantly different from each other on day 26 after LPS treatment (Figure 8B, insert).

**Figure 9:**
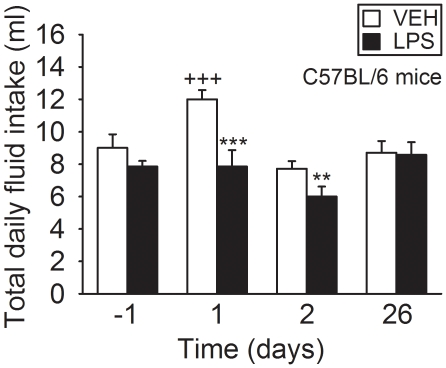
Effect of vehicle (VEH) and LPS (0.83 mg/kg injected IP) on the total daily intake of fluid in singly housed C57BL/6 mice. The graph shows the absolute daily intake of fluid on the day before treatment (day -1) and on days 1, 2 and 26 post-treatment. The values are means+SEM, n = 7. ** *P*<0.05, *** *P*<0.01 versus vehicle on the same day, +++ *P*<0.01 versus vehicle on all other days.

Further analysis demonstrated that the total intake of fluid significantly increased during day 1 after vehicle treatment but otherwise stayed constant during the 26-day observation period post-treatment (Figure 9). In contrast, the total consumption of fluid did not significantly change during the 26-day observation period after LPS treatment, but during days 1 and 2 post-treatment was significantly lower than after vehicle treatment (Figure 9).

## Discussion

### Behavioral analysis of female mice

The present data show that a single IP injection of LPS is able to induce long-term depression-like behavior in mice, an effect that depends on genetic background (strain) and, in part, on social environment. The dose of LPS used here (0.83 mg/kg) was identical to that used previously in the examination of its impact on depression-like behavior [12], [14]. The current study was carried out with female mice because affective disorders are more prevalent in women than in men [17], a situation that need be reflected in the design of translational animal experiments [18]. We did not monitor the estrous cycle in order to avoid additional stress which may influence the behavior of the animals. On the one hand, stress can prolong the estrous cycle [30] while, on the other hand, the estrous cycle can modify neural responses to endotoxin [31], depression-like behavior [32], [33] and circulating corticosterone [34]. Although being aware of these limitations, we consider it unlikely for a number of reasons that our data were significantly confounded by these factors. First, the experiments were carried out in the strict absence of male mice. Second, we have previously found that the estrous cycle is largely synchronized within the same and across different cages [35]. Third, the effects of LPS and its vehicle were examined in parallel under identical housing conditions. Fourth, the coefficient of variation of the data obtained in female mice did not appear anomalous in comparison to data obtained in male mice [36].

### Short-term behavioral effects of LPS

The short-term effect of LPS on behavior in the FST was evaluated because it may be of relevance to its long-term effect. It has previously been shown that singly housed male CD1 mice exhibit enhanced depression-like behavior in the FST 24 h post-LPS, as revealed by an increase in the immobility time at the expense of swimming time [12]. In the current study with female CD1 mice, an increase in depression-related behavior 1 day post-LPS was seen only under group housing conditions. In C57BL/6 mice, to the contrary, depression-like behavior was markedly enhanced by LPS only under single housing conditions. In addition, the behavioral profile of the depressogenic effect of LPS in C57BL/6 mice (prolonged immobility time, shortened swimming and climbing time) differed from that of CD1 mice (prolonged immobility time, shortened swimming time). The finding that, in group-housed C57BL/6 mice, LPS reduced immobility and enhanced climbing could be related to the apparent ability of vehicle to increase immobility and decrease climbing.

In interpreting these diverse observations made 1 day post-treatment several factors need be considered. First, the acute effect of LPS on behavior in the FST may rather be related to the transient sickness response, which can last for up to 2 days [37], than reflect a truly depressogenic effect [37]. Second, affective behavior in group-housed animals may be influenced by strain-related empathy among the cage mates [38]–[40] under the distress caused by LPS. Third, social isolation of mice can, on the one hand, increase the LPS-evoked sickness behavior [22] and, on the other hand, enhance depression-like behavior [25], [41]–[43]. Thus, the acute effect of LPS on the behavior in the FST under different housing conditions is a function of multiple interactive factors.

### Long-term depression induced by LPS

The major advance put forward by the present study is the discovery that LPS is able to induce long-term depression-like behavior in a strain-related manner. While the acutely depressogenic effect of LPS in group-housed CD1 mice had largely waned 4 weeks post-treatment, long-term depression in C57BL/6 mice was induced whether or not there was an acutely depressogenic effect. Importantly, the behavioral profile of the LPS-evoked long-term depression in group-housed C57BL/6 mice (prolonged immobility time, shortened swimming time) differed from that in singly housed C57BL/6 mice (prolonged immobility time, shortened climbing time).

The profile of the LPS-induced behavioral changes in the FST is worth noting because antidepressants that enforce serotonergic transmission increase primarily swimming behavior, while antidepressants that enhance noradrenergic transmission increase predominantly climbing behavior [15], [43]. In analogy, there is reason to speculate that the long-term depression-like behavior caused by LPS in group-housed C57BL/6 mice involves serotonergic but not noradrenergic pathways, while the depression-like behavior induced by LPS in singly housed C57BL/6 mice depends on noradrenergic but not serotonergic circuits. It would hence appear that the neurochemical substrates whereby LPS alters depression-like behavior in the FST differ with housing conditions. This instance provides a lead for targeted studies into the neurochemical mechanisms of immune challenge-evoked disturbances of affective behavior. The studies need to take account of particular characteristics of monoaminergic transmission in the brain of C57BL/6 mice. While C57BL/6 mice, like CD1 mice, are sensitive to the antidepressant effect of desipramine, a preferential norepinephrine reuptake inhibitor, in the FST [44], the serotonin transporter of C57BL/6 mice contains 2 non-synonymous single nucleotide polymorphisms that code for a reduced reuptake function and a reduced immobility time in the tail suspension test [16].

The prolonged depression-like behavior in LPS-treated C57BL/6 mice as observed in the FST was confirmed with the sucrose preference test in an independent cohort of animals. The two tests address different aspects of depression. While the FST measures behavioral despair [15], the sucrose preference test assesses anhedonia, a decrease in the ability to experience pleasure [12], [45], [46]. LPS exerted two distinct time-related effects on sucrose preference in singly housed C57BL/6 mice. One day post-treatment, there was a pronounced reduction of sucrose preference, an effect that waned during the subsequent days and had previously been noted in CD1 mice [12]. Twenty-six days post-treatment, however, the sucrose preference was found to be attenuated in LPS-treated C57BL/6 mice, which attests to a long-term effect of LPS to induce anhedonia-like behavior.

### Short-term and long-term effects of LPS on circulating interleukin-6 and corticosterone

The LPS-induced changes in body weight did not provide any explanation for its strain-dependent impact on affective behavior in the short and long term. The initial decrease in body weight is part of the transient “sickness response” to LPS, which includes anorexia and a reduction of locomotion and exploration [3], [6], [11], [47]. The sickness response arises from LPS-evoked induction of proinflammatory cytokines, including interleukin-6, that signal to the brain via a neural and endocrine route [6], [11], [22]. Our finding that LPS increased the post-FST plasma levels of interleukin-6 1 day but not 4 weeks post-treatment indicates that LPS, but not the FST, was responsible for the rise of this cytokine. The observation that circulating interleukin-6 in C57BL/6 mice increased to a much larger extent than in CD1 mice suggests that C57BL/6 mice are more sensitive to immune stress than CD1 mice. It remains to be shown whether the pronounced induction of interleukin-6 in C57BL/6 mice has any bearing on the long-term depression-like behavior induced by LPS in this mouse strain.

LPS and proinflammatory cytokines are able to stimulate the HPA axis as shown by a rise of circulating glucocorticoid levels [4]–[6], [22]. In the present study, the plasma levels of corticosterone measured 30 min post-FST do not represent basal levels of the glucocorticoid but reflect levels that are elevated by the stress of the FST procedure [36]. Single housing is stressful for female mice [48] and results in a reduction of the basal levels of circulating corticosterone [25]. Likewise, the post-FST levels of corticosterone measured 28 days after vehicle/LPS treatment were lower under single housing than under group housing conditions. We do not think, however, that the LPS-evoked long-term depression-like behavior in C57BL/6 mice arises primarily from a change in the function of the HPA axis, although two distinct alterations in the dynamics of the HPA axis were observed. First, LPS enhanced the FST-evoked increase in circulating corticosterone 1 day post-treatment independently of strain and housing conditions, whereas 4 weeks post-treatment this effect of LPS was largely gone. This observation is in keeping with the contention that the HPA axis undergoes prolonged desensitization after immune challenge [49]. Second, the post-FST plasma levels of corticosterone measured in C57BL/6 mice 28 days post-treatment were in general significantly lower than 1 day post-treatment. This observation may reflect a particular trait of C57BL/6 mice to respond to experimental interventions by a long-term change in HPA axis dynamics. Elucidation of the full impact of the HPA axis on LPS-evoked long-term changes in affective behavior will involve analysis of basal and stress-related levels of corticosterone and of the time course of the HPA axis response to stress.

### Conclusion

The current study demonstrates that immune challenge with LPS is able to induce prolonged depression-like behavior. Importantly, this effect depends on genetic background (strain). It has been noted before that the impact of immune challenge on affective behavior need be analyzed with respect to background conditions [22]. The availability of an experimental model of long-term depression-like behavior after acute immune challenge will aid the elucidation of the pathophysiology of immune system-related affective disorders and of the epigenome that is controlled by social environment and immune system activity [50].

## Supporting Information

Text S1 **Detailed Results of the Statistical Analysis of the Data.**
(DOC)
