# Supplementary material for: Prolonged Depression-Like Behavior Caused by Immune Challenge: Influence of Mouse Strain and Social Environment
Source: PLoS One. 2011 Jun 6;6(6):e20719. doi: 10.1371/journal.pone.0020719 (PMC3108969; doi:10.1371/journal.pone.0020719)
Supplement: Text S1 — Detailed Results of the Statistical Analysis of the Data. (DOC) [file pone.0020719.s001.doc]

**SUPPORTING INFORMATION**

**Prolonged depression-like behavior caused by immune challenge: influence of mouse strain and social environment**

Evelin Painsipp^1^, Martin J. Köfer^1^, Frank Sinner^2^, Peter Holzer^1^

^1^Research Unit of Translational Neurogastroenterology, Institute of Experimental and Clinical Pharmacology, Medical University of Graz, Graz, Austria

^2^Health - Institute for Biomedicine and Health Sciences, Joanneum Research, Graz, Austria

This file describes the detailed results of the statistical analysis of the data presented in Figures 2 – 9 of the main manuscript.

**Effect of LPS, relative to vehicle, on body weight (Figures 2 and 3)**

Two-way ANOVA for repeated measures showed that the body weight of singly housed CD1 mice (Figure 2A) recorded on days 0 and 1 differed with time (*F*_(1,14)_ = 20.80, *P* < 0.001) but not treatment (*F*_(1,14)_ = 0.31, *P* = 0.58), but with a significant interaction between these factors (*F*_(1,14)_ = 24.20, *P* = 0.001). Similarly, the body weight of group-housed CD1 mice (Figure 2A) measured on days 0 and 1 varied with time (*F*_(1,14)_ = 12.33, *P* = 0.003) but not treatment (*F*_(1,14)_ = 1.24, *P* = 0.29), with a significant interaction between these factors (*F*_(1,14)_ = 16.46, *P* = 0.001).

Comparison of the body weight of singly housed C57BL/6 mice (Figure 3A) recorded on days 0 and 1 by two-way ANOVA for repeated measures revealed a significant effect of time (*F*_(1,13)_ = 122.30, *P* < 0.001) and treatment (*F*_(1,13)_ = 9.95, *P* = 0.008), and a significant interaction between these factors (*F*_(1,13)_ = 141.26, *P* < 0.001). An analogous result was obtained in group-housed C57BL/6 mice (Figure 3A) in which the body weight differed both with time (*F*_(1,14)_ = 307.12, *P* < 0.001) and treatment (*F*_(1,14)_ = 5.12, *P* = 0.04), and there was a significant interaction between these factors (*F*_(1,14)_ = 267.70, *P* < 0.001).

Analysis of the body weights recorded on days 0 and 28 disclosed a significant difference with time in both singly housed (*F*_(1,14)_ = 19.18, *P* = 0.001) and group-housed (*F*_(1,14)_ = 9.79, *P* = 0.007) CD1 mice (Figure 2B), whereas there was no significant effect of the factor treatment and no significant interaction between the factors time and treatment. A similar observation was made in C57BL/6 mice (Figure 3B) in which the body weight taken on days 0 and 28 varied with time in both singly housed (*F*_(1,14)_ = 100.84, *P* < 0.001) and group-housed (*F*_(1,14)_ = 33.99, *P* < 0.001) animals. There was, however, no significant effect of the factor treatment and no significant interaction between the factors time and treatment.

**Effect of LPS, relative to vehicle, on the behavior of CD1 mice in the FST 1 and 28 days post-treatment (Figure 4)**

The duration of immobility and climbing recorded in singly housed CD1 mice 1 and 28 days post-treatment was independent of the factors time (1 day versus 28 days post-treatment) and treatment (LPS versus vehicle) as shown in Figure 4A,C. In contrast, the duration of swimming in singly housed CD1 mice varied with treatment (*F*_(1,28)_ = 16.92, *P* < 0.001) but not time, and there was no significant interaction between the factors time and treatment (Figure 4B).

In group-housed CD1 mice, the duration of immobility (Figure 4A) recorded 1 and 28 days post-treatment differed with the factors time (*F*_(1,28)_ = 5.30, *P* = 0.03) and treatment (*F*_(1,28)_ = 12.63, *P* = 0.001), with a significant interaction between these factors (*F*_(1,28)_ = 3.48, *P* = 0.07). While the duration of climbing was independent of the factors time and treatment (Figure 4C), the duration of time spent swimming by group-housed CD 1 mice (Figure 4B) varied with time (*F*_(1,28)_ = 4.28, *P* = 0.05) and treatment (*F*_(1,28)_ = 41.95, *P* < 0.001), with a significant interaction between these factors (*F*_(1,28)_ = 3.98, *P* = 0.06).

**Effect of LPS, relative to vehicle, on the behavior of C57BL/6 mice in the FST 1 and 28 days post-treatment (Figure 5)**

In singly housed C57BL/6 mice, there was an effect of treatment (vehicle versus LPS) on all three parameters assessed in the FST. Specifically, the duration of immobility recorded 1 and 28 days post-treatment differed with treatment (*F*_(1,28)_ = 21.43, *P* < 0.001) but not time, without a significant interaction between these two factors (Figure 5A). The duration of swimming (Figure 5B) varied with both treatment (*F*_(1,28)_ = 7.04, *P* = 0.01) and time (*F*_(1,28)_ = 3.13, *P* = 0.09), although there was no significant interaction between these factors. With regard to the time of climbing (Figure 5C), two-way ANOVA disclosed an effect treatment (*F*_(1,28)_ = 12.51, *P* = 0.001) but failed to reveal an effect of time and a significant interaction between these factors.

Two-way ANOVA of the duration of time spent immobile by group-housed C57BL/6 mice (Figure 5A) showed a significant interaction between the factors time and treatment (*F*_(1,28)_ = 20.13, *P* < 0.001), although there was no significant effect of treatment and time. A similar result was obtained with regard to the duration of climbing (Figure 5C) which did not significantly differ in the factors treatment and time, while a significant interaction between these factors (*F*_(1,28)_ = 5.28, *P* = 0.03) was revealed. The duration of swimming recorded in group-housed C57BL/6 mice (Figure 5B) varied with treatment (*F*_(1,28)_ = 11.37, *P* = 0.002) but not time, but with a significant interaction between these factors (*F*_(1,28)_ = 7.49, *P* = 0.01).

**Effect of LPS, relative to vehicle, on circulating corticosterone levels (Figure 6)**

Two-way ANOVA of the post-FST plasma levels of corticosterone in singly housed CD1 mice measured 1 and 28 days after treatment with LPS or its vehicle demonstrated an effect of treatment (*F*_(1,28)_ = 8.17, *P* = 0.008) but not time, without a significant interaction between these factors (Figure 6A). Similarly, the plasma concentrations of corticosterone in group-housed CD1 mice differed with treatment (*F*_(1,28)_ = 5.05, *P* = 0.03) but not time, without a significant interaction between these factors (Figure 6A).

In singly housed C57BL/6 mice (Figure 6B), the plasma levels of corticosterone levels depended on treatment (*F*_(1,27)_ = 14.19, *P* < 0.001) and time (*F*_(1,27)_ = 38.01, *P* < 0.001), and there was a significant interaction between these factors (*F*_(1,27)_ = 13.60, *P* < 0.001). Evaluation of the data obtained in group-housed C57BL/6 mice (Figure 6B) disclosed an effect of treatment (*F*_(1,27)_ = 22.42, *P* < 0.001) and time (*F*_(1,27)_ = 40.07, *P* < 0.001) and a significant interaction between these factors (*F*_(1,27)_ = 8.18, *P* = 0.008).

The plasma concentrations of corticosterone measured post-FST 28 days after vehicle/LPS treatment in singly housed mice were lower than in the respective group-housed animals (CD1 mice: *F*_(1,28)_ = 21.04, *P* < 0.001; C57BL/6 mice: *F*_(1,28)_ = 7.80, *P* = 0.009) but did not differ with regard to treatment (Figure 6A,B).

**Effect of LPS, relative to vehicle, on circulating interleukin-6 levels (Figure 7)**

Two-way ANOVA of the post-FST plasma concentrations of interleukin-6 in singly housed CD1 mice measured 1 and 28 days after treatment with LPS or its vehicle (Figure 7A) revealed an effect of treatment (*F*_(1,28)_ = 6.27, *P* = 0.02) and time (*F*_(1,28)_ = 8.22, *P* = 0.008) and a significant interaction between these factors (*F*_(1,28)_ = 5.86, *P* = 0.02). Similar data were obtained in group-housed CD1 mice (Figure 7A) in which the interleukin-6 levels differed with treatment (*F*_(1,28)_ = 5.12, *P* = 0.03) and time (*F*_(1,28)_ = 4.37, *P* = 0.05), with a significant interaction between these factors (*F*_(1,28)_ = 4.96, *P* = 0.03).

The circulating levels of interleukin-6 in singly housed C57BL/6 mice (Figure 7B) varied with treatment (*F*_(1,27)_ = 56.85, *P* < 0.001) and time (*F*_(1,27)_ = 55.81, *P* < 0.001), and there was a significant interaction between these factors (*F*_(1,27)_ = 57.35, *P* < 0.001). When the plasma levels of interleukin-6 in group-housed C57BL/6 mice (Figure 7B) were evaluated, two-way ANOVA likewise disclosed an effect of treatment (*F*_(1,28)_ = 36.73, *P* < 0.001) and time (*F*_(1,28)_ = 37.04, *P* < 0.001) and a significant interaction between these factors (*F*_(1,28)_ = 38.23, *P* < 0.001).

**Effect of LPS, relative to vehicle, on sucrose preference and total water intake in singly housed C57BL/6 mice (Figures 8 and 9)**

The effect of LPS, relative to vehicle, on sucrose preference over plain water in singly housed C57BL/6 mice was analyzed with two-way ANOVA for repeated measures taken during days -1 – 27 post-treatment (Figure 8A,B). The results demonstrated that in vehicle-treated mice (Figure 8A) there was an effect of treatment (*F*_(1,12)_ = 389.22, *P* < 0.001) and time (*F*_(2.08,24.92)_ = 5.65, *P* = 0.009) and a significant interaction between these factors (*F*_(2.08,24.92)_ = 12.27, *P* < 0.001). In LPS-treated mice (Figure 8B) there was likewise an effect of treatment (*F*_(1,12)_ = 64.99, *P* < 0.001) but not time, and a significant interaction between these factors (*F*_(1.69,20.27)_ = 13.41, *P* < 0.001). When the total intake of fluid following treatment of C57BL/6 mice with LPS or its vehicle (Figure 9) was analyzed, an effect of treatment (*F*_(1,12)_ = 7.46, *P* = 0.02) and time (*F*_(3,36)_ = 8.65, *P* < 0.001) and a significant interaction between these factors (*F*_(3,36)_ = 3.94, *P* = 0.02) was disclosed.
